# Supplementary material for: Effect of Sugar- and Polyphenol-Rich, Diluted Cloudy Apple Juice on the Intestinal Barrier after Moderate Endurance Exercise and in Ultra-Marathon Runners
Source: Nutrients. 2024 Apr 30;16(9):1353. doi: 10.3390/nu16091353 (PMC11085185; doi:10.3390/nu16091353)
Supplement: Supplementary file 1 [file nutrients-16-01353-s001.zip › nutrients-2950089-supplementary.pdf]

Supplemental Table S1. Results of the 24-hour recalls of the participants.

Nutrients	24-hour recall
Energy (kcal/day)	2504 ± 160
Carbohydrates (E%)	48.4 ± 2.4
Fat (E%)	33.7 ± 2.7
Protein (E%)	17.4 ± 1.2
Fiber (g)	35.9 ± 3.2

Values are means ± SEM. E%: percentage of energy.

Supplemental Table S2. Energy and macronutrient composition of the standardized meal served to the participants on the evening before the intervention day.

Nutrients	24-hour recall
Energy (kcal)	866 ± 51
Carbohydrates (E%)	56.5 ± 0.1
Fat (E%)	30.2 ± 0.2
Protein (E%)	14.0 ± 0.2
Fiber (g)	12.4 ± 0.5

Values are means ± SEM. E%: percentage of energy.
